# Mammalian Target of Rapamycin: Its Role in Early Neural Development and in Adult and Aged Brain Function

**DOI:** 10.3389/fncel.2016.00157

**Published:** 2016-06-16

**Authors:** Carla Garza-Lombó, María E. Gonsebatt

**Affiliations:** Departamento de Medicina Genómica, Instituto de Investigaciones Biomédicas, Universidad Nacional Autónoma de México, MéxicoMéxico

**Keywords:** mTOR, central nervous system, CNS development, adult brain, aging CNS

## Abstract

The kinase mammalian target of rapamycin (mTOR) integrates signals triggered by energy, stress, oxygen levels, and growth factors. It regulates ribosome biogenesis, mRNA translation, nutrient metabolism, and autophagy. mTOR participates in various functions of the brain, such as synaptic plasticity, adult neurogenesis, memory, and learning. mTOR is present during early neural development and participates in axon and dendrite development, neuron differentiation, and gliogenesis, among other processes. Furthermore, mTOR has been shown to modulate lifespan in multiple organisms. This protein is an important energy sensor that is present throughout our lifetime its role must be precisely described in order to develop therapeutic strategies and prevent diseases of the central nervous system. The aim of this review is to present our current understanding of the functions of mTOR in neural development, the adult brain and aging.

## Introduction

The mammalian target of rapamycin (mTOR) is a serine/threonine kinase that is involved in the control of cell growth and proliferation. This kinase integrates signals triggered by different stimuli such as variations in the amino acid supply, changes in the cellular energy state, and by receptors for various hormones and growth factors and in the brain by transduction of neurotransmitters and neurotrophin signals (Harris and Lawrence, 2003; Gal-Ben-Ari et al., 2012; Burket et al., 2015). Several studies have shown that mTOR participates in multiple functions of the brain. Its activation contributes to processes involved in synaptic plasticity and metabolic regulation (Garelick and Kennedy, 2011). Additionally, mTOR participates in key processes during neural development, particularly in axon and dendrite development, neuron differentiation, and gliogenesis. Furthermore, consistent with its role as a nutrient and growth factor sensor, decreased mTOR signaling reduces aging and thereby extends lifespan (Cornu et al., 2013). The aim of this review is to present our current understanding of the functions of mTOR in neural development, the adult brain and aging.

## The mTOR Multi-Protein Complex and Signaling Pathways

Metazoans have a single *TOR* gene, whereas yeast has two genes, *TOR1* and *TOR2*. In mammals, the *mTOR* gene (also known as *FRAP*, *RAFT1*, or *SEPT*) encodes a protein of 2549 amino acids with 42 and 45% sequence identity to yeast TOR1 and TOR2, respectively (Harris and Lawrence, 2003).

mTOR exists in two distinct multi-protein complexes; mTOR complex 1 (mTORC1) which is sensitive to rapamycin and consists of the proteins: regulatory associated protein of mTOR (RAPTOR), 40 kDa pro-rich AKT1 substrate 1 (PRAS40), mammalian lethal with SEC13 protein 8 (mLST8), and DEP domain-containing mTOR-interacting protein (DEPTOR). On the other hand, mTOR complex 2 (mTORC2) is relatively insensitive to rapamycin and consists of rapamycin-insensitive companion of mTOR (RICTOR), pro-rich protein 5 (PRR5, also known as PROTOR), DEPTOR, mLST8, and stress-activated MAP kinase-interacting protein 1 (mSIN1). However, prolonged treatment with rapamycin can also inhibit mTORC2, most likely due to the progressive sequestration of mTOR pools by rapamycin-FK506-binding protein (FKBP12; Sarbassov et al., 2005; Weber and Gutmann, 2012).

Rapamycin is an antifungal agent originally isolated from a strain of the soil bacterium *Streptomyces hygroscopicus.* It has potent immunosuppressive and antiproliferative properties and forms an inhibitory complex with its intracellular receptor, FKBP12, which binds to the C-terminus of TOR proteins, thereby inhibiting TOR activity (Weber and Gutmann, 2012). Because of the promising therapeutic potential of rapamycin, several rapamycin analogs have been synthesized to improve its pharmacokinetic properties (Tsang et al., 2007).

Nutrients, energy, stress, oxygen levels, and growth factors are among the major upstream signaling inputs for mTORC1. In this regard, the canonical mTORC1 pathway begins with the activation of tyrosine kinase receptors (Trk) through the induction of phosphoinositide-3-kinase (PI3K) and protein kinase B (PKB, also known as Akt) and includes the phosphorylation and inhibition of tuberous sclerosis complex proteins, hamartin (TSC1) and tuberin (TSC2). The TSC1/TSC2 complex acts as a GTPase-activating protein (GAP) for Ras homolog enriched in brain (Rheb). Subsequently, the increase in GTP-bound Rheb stimulates mTORC1 (Sarbassov et al., 2005; Weber and Gutmann, 2012).

Mitogens can also activate mTOR independently of Akt through phospholipase D (PLD) as well as through the extracellular signal-regulated kinase (ERK)–mitogen-activated protein kinases (MAPK) pathway (Jaworski and Sheng, 2006). The cellular energy levels (amino acid concentration) also require Rheb to activate mTORC1, however, instead of through the tuberous sclerosis complex (TSC), the signal is transduced via the Ras-related GTPase (Rag) complex, a heterodimer small GTPase-containing RagA or B with RagC or D. The increase in the amino acids concentration, induces the movement of mTORC1 to the lysosomal membranes where the Rag complex resides. The complex “Ragulator” consisting of Rag GTPase-interacting proteins (p18, p14, and MP1) has been identified in the interaction with the Rag GTPases and its consequent localization to the lysosome (Sancak and Sabatini, 2009; Kim and Guan, 2011).

Under conditions of energy deprivation that increase the AMP/ATP ratio, AMP-activated protein kinase (AMPK) becomes active and phosphorylates TSC2 to stimulate its GAP activity, therefore inhibiting Rheb and presumably mTORC1. Oxygen sensing by the mTORC1 pathway also requires the TSC1/2 complex, but it functions through a distinct mechanism that involves the hypoxia-inducible factor-dependent expression of regulated in development and DNA damage responses 1 (REDD1) and REDD2, two growth regulators (Sarbassov et al., 2005).

It has long been known that translational machinery operates in neuronal dendrites and synapses, and that local protein synthesis and its regulation is important for diverse processes of the central nervous system (CNS), e.g., memory and synaptic plasticity (Taha et al., 2013). In neurons, neurotransmission modulates the translation machinery, however, all the components of this delicate processes have not been identified yet. mTORC1 is a key downstream molecule in a signaling cascade beginning with the transduction of neurotransmitters and neurotrophin signals. Among the receptors found to activate mTORC1 in neurons, we can mention the muscarinic acetylcholine receptors, AMPA receptors, the glutamate metabotropic receptors (mGlu1/5), the dopaminergic D1 and D3 receptors, the opioid receptor, the amino acid/glutamate T1R1–T1R3 receptors, the serotonin 5-HT6 receptor, the cannabinoid 1 receptor (CB1R), and the GABAB receptors (Gal-Ben-Ari et al., 2012; Bockaert and Marin, 2015). Burket et al. (2015) discusses different articles where *N*-methyl-D-aspartate receptor (NMDAR) activation is downregulating mTOR signaling activity.

The hallmark of mTORC1 activity is the stimulation of ribosome biogenesis, mRNA translation, nutrient metabolism, and autophagy inhibition. In this regard mTORC1 regulates protein synthesis through the phosphorylation and inactivation of a repressor of mRNA translation, eukaryotic initiation factor 4E-binding protein (4E-BP1), and through the phosphorylation and activation of the S6 kinase (S6K1). Thus, S6K1 or 4E-BP1 phosphorylation is often used as an *in vivo* readout of mTOR activity (Hay and Sonenberg, 2004; Sarbassov et al., 2005). Phosphorylation of 4E-BP1 by mTORC1 signals the onset of cap-dependent translation; it allows the binding of several important initiation factors, as well as the positioning of the 40S ribosomal subunit at the 5′ end of the mRNA to begin the process of translation (Harris and Lawrence, 2003).

Active S6K1 stimulates the translation of mRNAs containing unique 5′-terminal oligopyrimidine tracts (TOPs). TOP-containing mRNAs encode ribosomal proteins, elongation factors and other critical components of ribosome production. However, S6K1 activity is not obligatory for this important process: cells lacking S6K1 still actively translate TOP mRNAs in response to growth factor stimulation (Weber and Gutmann, 2012). Furthermore, S6K regulates both the initiation and elongation phases of translation. It phosphorylates and inactivates the eukaryotic elongation factor 2 kinase (eEF2K) resulting in the dephosphorylation of the eukaryotic elongation factor 2 (eEF2) which mediates the translocation step of elongation (Wang et al., 2001; Taha et al., 2013). eEF2K is also named as calcium/calmodulin (CaM)-dependent protein kinase III (CaMKIII) because it can be activated by elevated levels of calcium and binding of CaM. In dendrites, this process depends on glutamate signaling and NMDAR activation, which suggests a complex regulation of the elongation step, dependent on particular cellular conditions (Taha et al., 2013; Heise et al., 2014).

**FIGURE 1:**
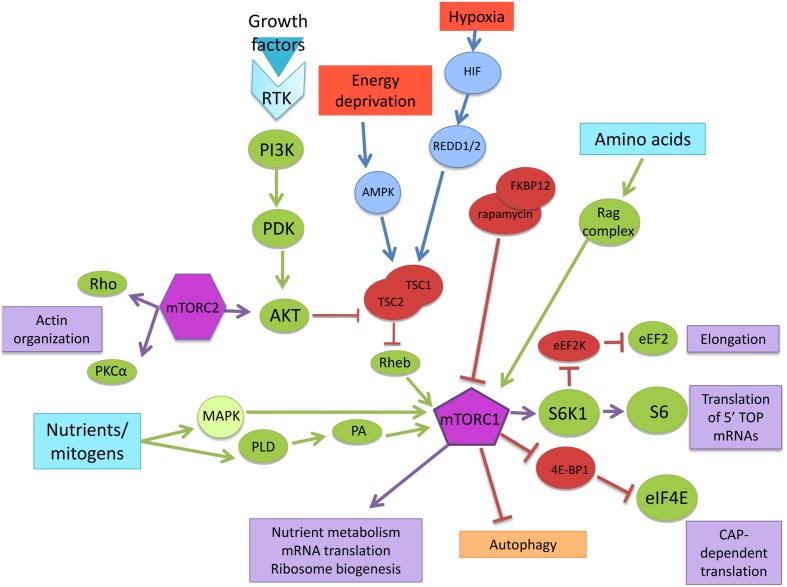
**mTOR signaling pathway.** Nutrients/mitogens, growth factors, energy, oxygen levels, and amino acids are the major upstream signaling inputs for mTORC1 via different signaling pathways. Through downstream effectors, mTOR controls nutrient metabolism, mRNA translation, and ribosome biogenesis.

Besides, mTORC2 plays an important role in Akt activation. Akt itself possesses pleiotropic cellular effects, regulating events including metabolism, survival, and proliferation. In addition to Akt, mTORC2 also regulates the actin cytoskeleton through unknown mechanisms that involve protein kinase C alpha (PKCα) and Rho. Evidence also points to mTORC2 as a key player in mRNA translation processes that have been isolated in polysomes and associated with individual ribosomal proteins (Sarbassov et al., 2005; Weber and Gutmann, 2012). Inactivation of mTORC2, but not mTORC1, conditional knockout (CKO) mice disrupted motor coordination early in life. The Purkinje cells using of these mice showed developmental alterations in the processes of climbing fiber elimination and dendritic self-avoidance (Angliker et al., 2015). Little is known about the upstream activating signals for mTORC2. Moreover, most of the activity of this protein complex has been studied in nervous system cancer cells such as glioblastomas where mTORC2 plays a central role in the metabolic reprogramming of this cancer pathology (Masui et al., 2015). The activity of mTORC2 in transformed cells is not discussed in this review (**Figure 1**).

## mTOR and Early CNS Development

Early CNS development is a complex, dynamic process that relies on a precisely orchestrated interplay of inductive signals and cellular migration. Building this complex and highly organized system involves the generation of a wide variety of specialized neural and non-neural cell types that must be placed at appropriate locations and with the right timing (Budday et al., 2015; Jiang and Nardelli, 2015).

Neurons, oligodendrocytes, astrocytes, and the ependymal lining of the central lumen are generated from a common source, the neuroepithelial cells that are part of the neural tube in early embryos (Kintner, 2002). Later, cell migration and extensive synapse formation are responsible for the initial establishment of the neural circuitry in the brain (Purves, 2012; Bury and Sabo, 2015). The first step in this process is to generate both axons and dendrites in the newly generated neurons. Axons must grow to reach the appropriate target cells and begin to make the synaptic connections that will form neural circuits (Barnes and Polleux, 2009; Purves, 2012).

Clinical evidence from developmental disorders that affect the CNS, including Lhermitte–Duclos disease (LDD), TSC, and neurofibromatosis type 1 (NF1) suggest that mTOR plays a key role in cell differentiation and growth control (Sandsmark et al., 2007; Crino, 2011). These inherited neurodevelopmental disorders and cancer syndromes are caused by mutations in genes that encode negative regulators of the mTOR-signaling pathway. The continued expression of mTOR in TSC affected individuals leads to benign overgrowths in multiple organs including brain. Neurological alterations such as epilepsy, autism, and learning disabilities are frequent among them and the administration of mTOR inhibitors result beneficial (Tee et al., 2016). Also, patients with germinal mutations in phosphatase and tensin homolog (*PTEN*; a negative modulator of mTOR) have been observed in 11 out of 31 LDD patients and associated with granular cell hypertrophy (Abel et al., 2005). Cognitive impairment is the most common neurological impairment in NF1 children which is frequently associated with neuropathological abnormalities (Gipson and Johnston, 2012).

### Neuron Differentiation

During embryonic development, the brain undergoes a dramatic transformation from a simple tubular structure to a highly convoluted shape. Neurulation is the process of forming the neural tube, which will become the brain and spinal cord. Soon after the neural tube forms, the forerunners of the major brain regions become apparent as a result of morphogenetic movements. Eventually, the anterior and posterior regions of the neural tube become the brain and spinal cord, respectively. Morphogenesis of the neural tube occurs in a specific spatiotemporal pattern along the length of the embryo (Sadler, 2005; Purves, 2012; Filas et al., 2013).

Neural stem cells (NSCs) in the early neural plate and tube and subsequently within each nascent brain region must follow instructions to differentiate into nerve cells specific to each region; balanced control of neural progenitor maintenance and neuron production is crucial for the establishment of functional neural circuits (Purves, 2012). *Mtor* null mice die before the differentiation of cortical neural progenitors into neurons (Ka et al., 2014). Thus, conditional mutant mTOR mice were developed to study functions and mechanisms of mTOR in developing brain. Conditional mutant mTOR mice had smaller brains with lower numbers of cells in the proliferating progenitors and cortical layer neurons. According to these authors an abnormal cell cycle progression disrupted the population of neural progenitors and thereby the progenitor self-renewal process (Ka et al., 2014).

Similar disruptive effects were observed in mice with a knock-in mutation in the PH domain of 3-phosphoinositide dependent protein kinase-1 (PDK1) and chick embryos treated with PI3K and TOR inhibitors exhibit deficient neuron production. Furthermore, rapamycin treatment repressed the expression of early neuronal differentiation genes, such as neurogenin 2 (*Ngn2*). Exposure to rapamycin can also disrupt coordination of cell cycle exit and progression of the neuronal differentiation program and interfere with the neuroepithelial organization (Fishwick et al., 2010). Rapamycin treatment in the early days of development resulted in a decrease in the number of progenitor stem cells available in the dentate gyrus of the hippocampus, as well as an impairment in the development of this region (Raman et al., 2013). Thus, TOR signaling levels may function as a checkpoint for the progression of differentiation, and appears to be essential for the production of NSCs and a proper development of the dentate gyrus of the hippocampus.

Decreasing mTORC1 activity with Rheb and RAPTOR knockdowns in neonatal NSCs in the subventricular zone (SVZ) prevented their differentiation resulting in reduced lineage expansion and aborted neuron production (Hartman et al., 2013). Also, the constitutive activation of the translational repressor 4E-BP1 had similar effects: it prevented hyperactive mTORC1 from inducing NSC differentiation and promoted self-renewal. By contrast, mTORC1-activated S6K1/S6K2 regulated NSC size but not differentiation, indicating different functions of 4E-BPs and S6K1/S6K2 in NSCs. These data demonstrated a crucial role of mTORC1 and 4E-BP for switching cap-dependent translation on and off in NSCs (Hartman et al., 2013).

On the other hand, Fu et al. (2012) generated CKO mice with selective deletion of the *Tsc1* gene in GABAergic interneuron progenitor cells, which have impaired growth and decreased survival. In this case, the cortical and hippocampal GABAergic interneurons of CKO mice were enlarged and showed increased mTORC1 signaling. Ectopic clusters of cells with increased mTORC1 signaling are also observed, suggesting impaired interneuron migration. Increased mTORC1 signaling may have negative effects on the CNS development, so regulatory proteins, such as TSC1, play important roles in keeping mTOR in check (Fu et al., 2012). In neuronal cultures from rat cortices, a triiodothyronine (T3)-dependent increase in the size of the soma in GABAergic neurons was mediated mainly by mTOR signaling. Both TrkB and mTOR signaling mediate the T3-dependent reduction of GABAergic axon extension, suggesting the existence of parallel regulatory pathways for T3-dependent changes (Westerholz et al., 2013).

When cortical cells from embryonic stages E13.5 and E16.5 were treated with transforming growth factor β (TGFβ) and insulin growth factor 1 (IGF1), IGF1 induced PI3K, Akt and mTORC1/mTORC2 primarily in E13.5-derived cells, resulting in proliferation, survival, and neuronal differentiation. At E16.5, TGFβ did not directly activate the Akt-mTOR pathway but required active PI3K-mTORC2-signaling to mediate the neuronal differentiation of cortical cells (Wahane et al., 2014).

The data presented in these studies suggest that mTOR plays an important role in the development of neuronal progenitors, controlling proliferation and differentiation in various regions of the CNS. It seems that mTOR may function as a checkpoint for the differentiation progression, while its hyperactivation does not inhibit but can affect interneuron migration and neuron differentiation.

### Axon and Dendrite Development

Once nerve cells have been generated, neurons must become interconnected to form the neural circuits. The first step in this process is to establish axons and dendrites in the newly generated neurons. Differentiation of axons and dendrites is a critical step in neuronal development (Cheng and Poo, 2012; Purves, 2012). Neurons typically form a single axon and multiple dendrites, which underlie the directional flow of information transfer in the CNS. Axons grow to reach appropriate target cells and begin to make the synaptic connections that will form neural circuits. Dendrites integrate synaptic inputs, triggering the generation of action potentials, making presynaptic contacts onto target cells. There are multiple signaling pathways underlying the establishment of axon-dendrite development (Barnes and Polleux, 2009; Purves, 2012). As in neuron differentiation, there are several studies that show that mTOR plays a pivotal role in both dendritic and axonal growth.

The pharmacological stimulation of the serotonin receptor subtype 7 (5-HT_7_R) using a highly selective agonist, LP-211, enhances neurite outgrowth in primary neuronal cultures derived from embryonic mouse brain, via ERK, cyclin-dependent kinase 5 (Cdk5), mTOR, and cell division control protein 42 homolog (Cdc42). However, the neurite elongation induced by the agonist stimulation of 5-HT_7_R is dependent on mTOR, because the outgrowth is completely inhibited by both rapamycin and torin 1, a specific ATP-competitive mTOR inhibitor (Speranza et al., 2015).

Methylcobalamin (MeCbl), a vitamin B12 analog, increases mTOR activity via the activation of Akt and promotes neurite outgrowth in cerebellar granule neurons while rapamycin decreased the effect of MeCbl on neurite outgrowth (Okada et al., 2011).

In a shRNA-induced selective knockdown of RAPTOR and RICTOR, it was shown that both mTORCs are needed for the presence of proper dendritic morphology of neurons, whether grown under basal culture conditions and treated with insulin or transfected with constitutively active PI3K. Neurons with knockdown of either RAPTOR or RICTOR produced fewer new dendrites, whereas the number of retracted dendrites remained relatively stable (Urbanska et al., 2012).

However, different outcomes have been reported with respect to the positive role of mTOR in neurite elongation and growth. The overexpression of the mammalian nicotinamide-adenine dinucleotide-dependent deacetylase sirtuin 1 (Sirt1) promoted neurite outgrowth and improved cell viability under both normal and stress conditions, such as nutrient deprivation or neurotoxic insult, in primary culture systems. Enhanced Sirt1 expression in neurons downregulated the protein levels and phosphorylation of mTOR. Correspondingly, rapamycin markedly improved neuronal cell survival in response to nutrient deprivation and significantly enhanced neurite outgrowth in wild-type mouse neurons. Sirt1 is activated in low energy availability promoting tissue repair processes, while mTOR is activated during high energy conditions (Garcia-Rodriguez et al., 2014). In this case, the above results suggest that mTOR could have negative effects on CNS development, so regulatory proteins such as Sirt1 play important roles in keeping mTOR in check (Guo et al., 2011).

Regarding axon and dendrite development, the studies presented here show that mTOR participates in the processes of neurite production, promoting outgrowth and elongation, as well as in maintaining proper dendritic morphology. On the other hand, Guo et al. (2011) showed that rapamycin also enhances neurite outgrowth. These apparently contradictory results could be interpreted in terms of spatiotemporal participation of mTOR in these processes, which is extremely tightly regulated. During embryonic development, the temporary silencing of some proteins has been reported as a switch between stages (Hirabayashi and Gotoh, 2010; Golbabapour et al., 2013). However, we must consider that most of these studies used *in vitro* models where cell type and culture conditions could greatly influence the results mainly in key regulators of cell metabolism such as Sirt1 and mTOR. Further *in vivo* studies are necessary to be able to confirm the role of mTOR in axon and dendrite development.

### Gliogenesis

Glia constitute the majority of the brain cells, they perform key functions vital to CNS physiology, including blood–brain barrier formation and maintenance, synaptogenesis, neurotransmission, and metabolic regulation. Glial cell generation starts late in embryonic stage. Regulation of neuron to glia switch involves complex neuron–glial interactions as well as spatiotemporal interplay of both cell-intrinsic factors and extracellular signals (Jiang and Nardelli, 2015; Molofsky and Deneen, 2015).

mTORC1 signaling pathway has a crucial function in the process of astrocyte differentiation (Lee da, 2015). Evidence from different reports show that the axon inhibitor Nogo-66 promotes the NSCs differentiation into the glial lineage via mTOR and the signal transducer and activator of transcription 3 (STAT3; Rajan et al., 2003; Wang et al., 2008; Cloetta et al., 2013). Similar to mTORC1, in NF1 genetically engineered mice and derivative NSC neurosphere cultures, was shown that hyperactivation of RICTOR containing mTORC2 activation also increases gliogenesis in the brainstem (Lee da et al., 2010).

Oligodendrocytes are the glial cells that generate CNS myelin; myelin is a multilamellar differentiation of the oligodendrocyte plasma membrane that sheaths axons to facilitate electrical conduction. Progression through the oligodendrocyte lineage is tightly regulated by a multitude of intrinsic and extrinsic cues, which control myelination both spatially and temporally during development and after demyelination. These signals include growth factors, protein kinases, and extracellular matrix molecules, all of which influence epigenetic modifications, transcriptional and translational regulation, and the actin cytoskeleton in oligodendrocytes (Zuchero and Barres, 2013; Bercury and Macklin, 2015).

Both *in vivo* and *in vitro* studies have demonstrated that the mTOR pathway is an essential mediator during oligodendrocyte development and that it can be associated to several external growth factors or hormones with known roles in oligodendroglia differentiation and myelination (Wood et al., 2013). For example, conditionally ablation of either RAPTOR or RICTOR in the oligodendrocyte showed that RAPTOR is a positive regulator myelination whereas RICTOR ablation has a modest positive effect on oligodendrocyte differentiation, and very little effect on myelination (Bercury et al., 2014; Bercury and Macklin, 2015). Purified oligodendrocyte progenitor cells (OPCs) treated with CB receptor agonists and antagonists, as well as with PI3K-Akt and mTOR inhibitors. Maximal phosphorylation of mTOR was obtained after 10 min stimulation with the agonist HU210, and this level of phosphorylation was sustained for 60 min. In contrast, incubation with the other agonists ACEA or JWH133 provoked transient mTOR phosphorylation that peaked at 2 min and then fell below the basal level (Gómez et al., 2011).

The roles of Rheb1 and mTORC1 in the mouse oligodendrocyte lineage were examined using separate *Cre* drivers to generate *Rheb1* or *mTor* CKO animals for OPCs as well as for differentiated and mature oligodendrocytes. Deletion of *Rheb1* in OPCs impairs the differentiation to mature oligodendrocytes, which involves mTORC1. This was accompanied by a reduction in OPCs exiting the cell cycle, similarly as during neuron differentiation (Zou et al., 2014).

mTOR is downstream of known activators of PI3K-Akt signaling and upstream of a number of targets important for regulating many aspects of oligodendrocyte differentiation and myelination, including nuclear transcriptional regulators, mediators of cytoskeletal organization and enzymes necessary for lipogenesis (Wood et al., 2013). The finding that both Rheb1 and mTORC1 are essential for early stage differentiation of OPCs to mature oligodendrocytes, which occurs during a narrow time window, reinforced its importance. Additionally, it seems that the endocannabinoids may be the extracellular signals that activate Akt and mTOR during oligodendrocyte differentiation; the activation of mTOR depended on the agonist used and the CB receptors activation.

## mTOR in the Adult CNS

As we mentioned in the previous section, during early postnatal life, the neural circuits process diverse types of information and build the main neural circuits. The sensory systems process information about the state of the organism and the environment, and the motor systems organize and generate actions, and associational systems link the sensory and motor components, providing the substrate for higher brain functions such as perception, attention, cognition, emotions, language, rational thinking, and consciousness (Purves, 2012).

Neurogenesis persists in adult mammals in specific brain areas known as neurogenic niches. Either physiological or pathological conditions can modulate the rate of proliferation of adult NSCs via the differentiation and fate determination of progenitor cells, as well as the survival, maturation, and integration of newborn neurons. Adult neurogenic niches can be conceptualized as remnants of embryonic signaling centers: they are the source of instructive signals that determine the fate of neighboring stem cells. Furthermore, these cells may be required for some learning and memory processes (Zhao et al., 2008; Urban and Guillemot, 2014).

### Learning, Memory, and Synaptic Plasticity

Learning-related changes include modulation of synaptic and non-synaptic ion channels and receptors, dendritic branching, spine density, and plasticity through genetic and epigenetic mechanisms (Sehgal et al., 2013; Stuchlik, 2014). Synaptic connectivity between neurons is a dynamic entity that is constantly changing in response to neural activity and other influences. At the shortest time scales, facilitation, augmentation, potentiation, and depression provide rapid but transient modifications in synaptic transmission (Klann and Dever, 2004; Purves, 2012; Bailey et al., 2015).

The biological processes mediating memory formation involve numerous tightly regulated molecular and cellular events. These include mRNA transcription, protein synthesis, mRNA and protein degradation, mRNA and protein trafficking, post-translational modifications such as phosphorylation and ubiquitination, and epigenetic modulation. Such processes can be brain hemisphere- and brain subregion-specific (Kandel, 2001; Middei et al., 2014; Rosenberg et al., 2014).

Long-lasting synaptic plasticity and memory rely on protein synthesis. Not surprisingly, TOR, one of the major controllers of translation, has been involved in important processes such as synaptic plasticity, memory and diverse types of behavior in various organisms, including mammals (Swiech et al., 2008).

As in early CNS development, mTOR also participates in processes such as synaptic plasticity, adult neurogenesis, learning, and memory. Its role depends on the regions of the CNS studied and the stimuli used. Here we describe the participation of mTOR in signaling pathways in the hippocampus, striatum, amygdala, medial prefrontal and auditory cortices.

#### Hippocampus

The hippocampus is essential for memory formation; in addition, its neuronal connections are highly ordered, and it is easy to identify specific populations of neurons and synapses. Here, we reviewed studies that suggest that mTOR participates in hippocampal neurogenesis, neurite growth and survival, and dendritic spine formation.

We have mentioned that Akt-mTOR participates in dendritic development. This signaling pathway was identified as an unexpected target of the gene disrupted-in-schizophrenia 1 (DISC1) regulating morphogenesis and dendritic development in new neurons in the adult mouse hippocampus (Kim et al., 2009). There is also evidence that insulin receptor (IR) regulates dendritic spine formation and excitatory synapse development in primary cultures of rat hippocampal neurons through the activation of the PI3K-Akt-mTOR signaling pathway, which in turn promotes Rac1-dependent actin cytoskeletal rearrangement and dendritic spine formation (Lee et al., 2011). Moreover, Ezh2 (methyltransferase of histone H3K27, enhancer of zeste homolog 2) regulates adult neurogenesis and preserves cognitive functions via the Akt-mTOR pathway (Zhang et al., 2014). The methyltransferase suppresses PTEN expression, promoting the activation of Akt-mTOR. The deletion of *Ezh2* in progenitor cells leads to a decrease in neuron production *in vivo*; more importantly, *Ezh2*-null mice showed impairments in spatial learning and memory, contextual fear memory, and pattern separation.

Similarly, the administration of ABG001 (tetradecyl 2,3-dihydroxybenzoate), a neuritogenic substance, enhanced the survival and neurite growth of newborn cells in adult hippocampal via TrkA receptor-triggered ERK and PI3K-Akt-mTOR signaling pathways, that leads to an enhanced preferential spatial cognitive function. The treatment of amyloid beta_25-35_ (Aβ_25-35_)-mice with ABG001 protected the survival and neurite growth of newborn cells by increasing TrkA receptor-induced phosphorylation of Akt and mTOR, which was accompanied by improved spatial cognitive performance (Zhou et al., 2014).

In cultured hippocampal neurons, mTORC1 activity tags synapses, allowing the RNA-stabilizing protein HuD to capture the Ca^2+^ calmodulin-dependent protein kinase II α (CaMKIIα) in a branch-specific manner, promoting site-specific and long-lasting forms of plasticity in the tagged branch. Thus, mTORC1 and HuD are good candidates to target the mRNAs coding for proteins required to strengthen neighboring synapses to facilitate late-stage plasticity (Sosanya et al., 2015).

Most importantly, Su et al. (2015) showed that the activation of mTOR pathway components was lower in the hippocampus of premature rats relative to full-term rats, and this was associated with poorer learning and memory performance. They showed that long-term consumption of a protein-rich diet can restore the impairment in learning and memory in pre-term rats via upregulation of mTOR-S6K1 signaling. In addition, mTOR activation and phosphorylation of S6K1 and 4E-BP1 in rat hippocampus was observed during a spatial learning paradigm. When mTORC1 was inhibited by chronic intra-cerebral ventricular infusion of rapamycin the phosphorylation of mTOR substrates were also inhibit as well as the learning-induced enhancement of protein synthesis and the acquisition of learning. These results show the activation of mTOR and its downstream targets during spatial learning in hippocampal pyramidal neurons (Qi et al., 2010).

There are several recent studies addressing the participation of mTOR and hormones in memory consolidation and learning. First, three different studies showed that the sex hormones estrogen and progesterone induced memory consolidation in the hippocampus. Estradiol induced the enhancement of object-recognition memory consolidation via PI3K-mTOR activation in the dorsal hippocampus (Fortress et al., 2013). Estradiol also modulated hippocampal synaptic plasticity by activating mTOR through a signaling pathway that involved TrkB activation, ERK phosphorylation, and calpain activation (Briz and Baudry, 2014). In young ovariectomized mice, bilateral dorsal hippocampal infusion of progesterone significantly increased the levels of ERK and S6K1. This activation was essential for the progesterone-induced facilitation of memory consolidation (Orr et al., 2012). These studies showed that rapid protein synthesis is necessary for these hormones to modulate the consolidation of hippocampal LTP and memory.

Also, the neurotrophin brain-derived neurotrophic factor (BDNF) is an upstream activator of mTOR during and after training to regulate GluR1 translation in hippocampal synaptic plasma membranes during IA (inhibitory avoidance) training memory consolidation (Slipczuk et al., 2009).

The markers for initiation of translation, including eukaryotic translation initiation factor 4E (eIF4E), 4E-BP1, ribosomal protein S6, and eIF4F cap-complex formation, undergo diurnal oscillations in the mouse hippocampus. This diurnal oscillation in translation initiation has been associated with increased activity of ERK1/2 MAPK and mTOR, and it was lost in memory-deficient mice lacking calmodulin-stimulated adenylyl cyclases 1 and 8. Disruption of circadian rhythms leads to loss of diurnal translation oscillation and causes memory deficits; moreover, inhibition of protein synthesis during the midday for 4 days post-training impairs memory persistence (Saraf et al., 2014).

The stimulation of the cannabinoid receptor CB1R by endogenous and exogenous cannabinoids can trigger the activation of the mTOR pathway and protein synthesis in the hippocampus. Contrary to what has been observed in other cases, this activation causes long-term memory impairment and amnesic-like effects, probably due to the fact that CB1R is mainly expressed in GABAergic interneurons. These findings suggest that the activation of mTOR through CB1R would contribute to an imbalance between the excitatory and inhibitory inputs in the hippocampus (Puighermanal et al., 2009).

Moreover, disruption of DISC1 function in adult-born dentate granule neurons is sufficient to cause several profound behavioral phenotypes, including pronounced learning and memory deficits, as well as clear anxiety and depression-like phenotypes. The knockdown of *Disc1* leads to increases in mTOR, indicating that this signaling abnormality is responsible for the cognitive and affective deficits. Rapamycin reversed these behavioral deficits even when associated neuroanatomical abnormalities persisted; remarkably, the rapamycin treatment, which rescued memory deficits in shRNA-DISC1 mice, caused memory deficits in control mice (Zhou et al., 2013).

Most studies also showed that mTOR is involved in both memory and learning consolidation processes, as well in the responses of various behavior to hormones, BDNF, and circadian changes. Furthermore, long-term memory deficits can be associated with an overactivation of the mTOR signaling pathway and an imbalance in protein synthesis, suggesting that mTOR requires mechanisms for tight spatiotemporal control of its expression.

#### Striatum

For motor control and learning, the basal ganglia, the motor cortex, and the cerebellum cooperate to enable movement; the striatum, part of the basal ganglia, plays a significant role in the learning processes of motor skilled tasks. Bergeron et al. (2014) analyzed whether mTOR activity is influenced or engaged during the execution of motor movement and motor learning. They showed that mTOR activity of the dorsal striatum is an important molecular step involved in learning consolidation during the acquisition of a complex motor skill in mice, but it is not related to motor abilities. These data are consistent with the studies in the hippocampus showing the role of mTOR in memory and learning consolidation.

Mice with disrupted mTORC2 signaling in the striatum exhibit altered striatal dopamine-dependent behaviors, such as increased basal locomotion, stereotypic counts, etc., by altering the D2R and ERK1/2 pathway (Dadalko et al., 2015).

#### Amygdala

The amygdala mediates neural processes that relate sensory experience with emotional significance, and it is also the site where learning about fearful stimuli occurs. It was found that rapamycin increases neuronal activity and anxiety-related behavior, impairs both consolidation and reconsolidation of an auditory fear memory, and produces impairment of IA memory. Given the importance of the amygdala in mood regulation, associative learning, and modulation of cognitive functions, it is important to consider the role of mTOR in this region; rapamycin can be used as a treatment for reducing the emotional strength of established traumatic memories analogous to those observed in acquired anxiety disorders, but it may also induce alterations in mood regulation.

In three different studies, Jobim et al. (2012a,b) and Pedroso et al. (2013) showed that infusion of rapamycin into the basolateral complex of the amygdala and hippocampus before and after a reactivation (retrieval) session produced IA memory impairment. These data showed that non-reinforced fear memory retrieval could lead to memory reconsolidation through a mechanism that requires protein synthesis and mTOR signaling in the amygdala and hippocampus.

The systemic administration of a single low dose of rapamycin led to enhanced neuronal activity in the amygdala and an increase in anxiety-related behaviors. The behavioral alterations correlated to enhanced amygdalar expression of KLK8 and FKBP51, proteins that have been implicated in the development of anxiety and depression (Hadamitzky et al., 2014). Moreover, systemic inhibition by rapamycin administration immediately or 12 h after either training or reactivation for auditory fear conditioning, blocks both consolidation and reconsolidation-like activities that contribute to the formation, retention, and maintenance of long-term memory. These data suggest that biphasic translational control through the mTOR pathway is normally required during the long-term formation and stabilization of memory through recurrent consolidation and reconsolidation-like events (Mac Callum et al., 2014).

#### Medial Prefrontal Cortex

The amygdala is connected with the medial prefrontal cortex (mPFC), a brain region that is involved in decision making, task switching, memory consolidation, and the retrieval of remote long-term memory. It was shown that the PI3K-Akt-mTOR signaling pathway is involved in the LTP and mPFC-dependent long-term trace fear memory (Sui et al., 2008). Another study in the mPFC demonstrated that overexpression of S6K1 produces antidepressant effects in the forced swim test without altering locomotor activity. Conversely, expression of dominant-negative S6K in the mPFC resulted in prodepressive behavior in the forced swim test and was sufficient to cause anhedonia in the absence of chronic stress exposure. These data demonstrate a critical role for S6K1 activity in depressive behaviors and suggest that pathways downstream of mTORC1 may underlie the pathophysiology and treatment of major depressive disorder (Dwyer et al., 2015).

These results suggest that fear regulation is mediated by connections from the mPFC to the amygdala, and it seems that mTOR participates in this relationship; in the mPFC, the animals learn to predict aversive events via mTOR, and in the amygdala, the memory is retained. However, mTOR also participates in avoiding anxiety and depression.

#### Auditory Cortex

The auditory cortex of mammals mediates particular aspects of auditory stimulus processing, task-specific performance, and learning; one report (Schicknick et al., 2008) showed that the memory required for the discrimination of complex sensory stimuli is controlled by dopaminergic activity via mTOR.

In the same way, the dopaminergic inputs to the gerbil auditory cortex regulate mTOR-mediated, protein-synthesis-dependent mechanisms, thereby controlling the consolidation of memory required for the discrimination of complex auditory stimuli (Schicknick et al., 2008). In a more recent work of this group, Reichenbach et al. (2015) tested the impact of local pharmacological activation of different D1/D5 dopamine receptor signaling modes in the auditory cortex and found differentially regulation of several protein profiles related to rearrangement of cytomatrices, energy metabolism, and synaptic neurotransmission in cortical, hippocampal, and basal brain structures. These results may be mTOR-mediated, which, in turn, might enhance the ability to synthesize plasticity-related proteins locally on demand and facilitate the consolidation of discrimination memory (Reichenbach et al., 2015).

## mTOR and CNS Aging

During normal aging, the brain suffers both morphological and functional modifications that affect dendritic trees and synapses, neurotransmission, circulation, and metabolism; these changes promote neurodegeneration, impair neurogenesis, and can be considered a cause of cognitive impairment and sensory and motor deficits in the elderly (Mariani et al., 2005; Sarlak et al., 2013). Mounting evidence, however, appears to implicate increased susceptibility to the long-term effects of oxidative stress, mitochondrial dysfunction and inflammatory insults as major contributing factors (Mariani et al., 2005). Understanding the mechanisms and the detailed metabolic interactions involved in the processes of normal and pathological neuronal aging and thus improving health is critical to increasing the quality of life in the elderly population, especially given the dramatic increase in the aging population worldwide (Toescu, 2005; Yang et al., 2014).

In recent years, the manipulation of nutrient-sensing and stress-response pathways has extended the lifespans of organisms from yeast to mammals. Growth-promoting cell programs may accelerate aging by generating metabolic by-products and by directly inhibiting the clearance of these by-products (Zoncu et al., 2011). mTOR is a prime target in the genetic control of aging, and evidence from genetic studies supports the view that mTOR may be a master determinant of lifespan and aging in yeast (Kaeberlein et al., 2005), worms (Vellai et al., 2003), flies (Bjedov et al., 2010), and mice (Harrison et al., 2009).

There is compelling evidence that cellular mechanisms and signaling pathways regulating brain aging and age-related neurodegenerative disorders are at least partially controlled by mTOR. The complex signaling networks underlying the age-related effects have not been fully elucidated. There are multiple reviews discussing the relationship between mTOR and CNS aging (Maiese et al., 2012; Sarlak et al., 2013; Jenwitheesuk et al., 2014; Perluigi et al., 2015); here, we describe recent studies on this topic.

One of the mechanisms that relate mTOR to aging is its participation in inhibiting autophagy. Autophagy is essential for removing damaged macromolecules and organelles from the cytoplasm and recycling amino acids (Mizushima et al., 2008). Studies suggest that autophagy declines with age and that this leads to an accumulation of damage, such as protein aggregates and degenerated mitochondria, that contribute to age-related cellular dysfunction (Cuervo, 2008).

A recent study using naked mole rats which live for up to 24 years, shows that they maintain high brain autophagy levels during the majority of their lifespan. Additionally, the p-mTOR/mTOR ratio showed a significant increase from the early to intermediate age group and a significant decrease from the intermediate age group to the old and oldest age groups (Triplett et al., 2015).

In mouse retinal pigment epithelium (RPE) explants and cultured human RPE cells, aged RPE cells contained more lysosome-associated mTOR and showed an increased response to amino acid stimulation. Increased mTORC1 activity caused a decreased rate of degradation of internalized photoreceptor outer segments. These data suggest that the Rag–Ragulator complex controls the lysosomal distribution of mTORC1 in RPE cells and may further exacerbate the lysosomal dysfunction of aged RPE (Yu et al., 2014).

The use of caloric restriction (CR) as a strategy to study mechanisms behind aging and age-associated diseases is based on evidence suggesting that CR can delay aging and protect the CNS from neurodegenerative disorders. Yang et al. (2014) used C57BL/6J mice with different diets and found that cognitive function declined with aging, especially after 12 months of age while CR ameliorated the age-dependent cognition deficit by deactivating mTOR and its upstream BDNF-Akt signaling in the hippocampus. Unexpectedly, they found a decline in mTOR signaling with aging, suggesting that other mediators play a more important role in regulating age-dependent autophagy than mTOR by itself in this model.

Dong and colleagues published two other studies using the CR strategy. C57BL/6 mice were randomly assigned to a NC group (standard diet), a CR group or a HC group (high-calorie diet) for 10 months. Activation of the mTOR/S6K1 and p62 signaling pathways was significantly upregulated in hippocampal neurons of mice in the HC and NC groups and ameliorated by the CR treatment. They also found that the HC diet and CR have opposing effects on learning and memory related to age. Different caloric intake may be an important way to accelerate or slow the progression of age-related neurodegenerative disorders (Dong et al., 2015a,b).

Furthermore, when the epigenetic changes in old mice with dietary restriction (DR) were analyzed, the histone methylation levels of H3K27me3, H3R2me2, and H3K79me3 in 22-month-old mouse brain were lower than in 3-month-old animals. However, either DR or rapamycin treatment prevented the age-induced losses of H3K27me3, H3R2me2, and H3K79me3. Moreover, DR and rapamycin each enhanced the levels of H3K18ac and H3K4me2. The level of H3K4me declined with age and was further diminished by either DR or rapamycin. The results from this study suggest that either DR or rapamycin can restore, at least partially, the age-related alterations in histone methylation levels (Gong et al., 2015).

Mild mitochondrial uncoupling is similar to the physiological energetic challenges, including exercise and intermittent fasting, that seem to protect the CNS from the effects of aging. The induction of mild mitochondrial uncoupling using 2,4-dinitrophenol (DNP) triggers a complex integrated cellular response in the brain that includes suppression of mTOR and insulin signaling, enhanced autophagy, and upregulation of cAMP response element-binding (CREB), changes that are known to play important roles in synaptic plasticity. Moreover, DNP treatment improves the performance of mice in a learning and memory task (Liu et al., 2015).

In terms of cognitive function and memory in the elderly, genome-wide screening showed a novel association of a polymorphism in the pro-apoptotic gene *FASTKD2* (fas-activated serine/threonine kinase domains 2) with better memory performance in older adults. Complementary analyses at the gene and pathway levels identified additional genome-wide significant associations with episodic memory and the genes *LARS2* (leucyl-tRNA synthetase 2, mitochondrial) and *mTor*, which along with *FASTKD240*, encode proteins with critical roles in mitochondrial function. These new findings identify potential targets to help improve risk stratification and therapeutic development in normal cognitive aging and dementia (Ramanan et al., 2015).

Adult neurogenesis is another process that decreases with age. This is mainly because of the reduction in the proliferation of active NSCs. As mice age, the activity of the mTOR signaling pathway decreases in the NSCs. Short-term treatment with ketamine significantly restored the proliferation of NSCs in the aged mice via mTOR signaling activity. Stimulating mTOR signaling revitalized the NSCs, restored their proliferation, and enhanced neurogenesis in the hippocampus of the aged brain. These data show the potential of mTOR for restoring neurogenesis in elderly individuals, but it is important to be aware that overstimulation of mTOR may also lead to other unwanted effects, such as stem cell depletion, aging, and carcinogenesis (Romine et al., 2015).

Also Paliouras et al. (2012) showed that mTOR is pivotal in determining proliferation versus quiescence in the adult forebrain niche. mTOR inhibition yielded a quiescence-like phenotype *in vitro*, while epidermal growth factor (EGF)-induced upregulation of mTOR activity enabled the reactivation of the quiescent SVZ niche within the aging brain. These findings reveal that the mTOR signaling pathway is a key regulator of neurogenesis in the adult and aging brain.

Finally, the deficiency of the core clock protein BMAL1 increased mTORC1 activity. A significant increase in phosphorylation of downstream targets of mTORC1 was observed at several time points in different tissues of *Bmal1–/–* mice. Treatment with rapamycin increased the lifespan of *Bmal1–/–* mice. The authors suggest that the circadian clock controls the activity of the mTOR pathway through BMAL1-dependent mechanisms and that this regulation is important for the control of aging and metabolism (Khapre et al., 2014).

The role of mTOR during aging seems to participate in more mechanisms than merely autophagy, and it will be challenging to untangle the role of mTOR in all these processes. The findings also depend on the model and the approach used. mTOR increases with age and inhibits autophagy, an important process in degradation of cellular debris, as well as impairing cognitive functions, and these effects can be ameliorated with a CR diet or with mTOR inhibitors such as rapamycin; in fact, rapamycin has been approved clinically for a variety of uses, including as an immunosuppressant and as an anticancer drug. Yang et al. (2014) report evidence of a decline in mTOR with aging, suggesting that there could be other longevity factors that can also regulate basal autophagy in the mouse hippocampus during normal brain aging.

mTOR is also regulated by the circadian rhythm and by mitochondrial uncoupling and is related to a polymorphism associated with better memory performance in humans. mTOR regulates processes such as epigenetic changes (histone methylation levels), neurogenesis and proliferation in various niches in the aging brain. Therefore, inhibiting mTOR to increase the lifespan should be undertaken with caution because this protein participates in pathways that are essential for health, and it is likely that alterations in mTOR activity could have negative effects throughout the entire system (**Table 1**).

**Table 1:** mTOR role and signaling components in the different stages of the CNS.

Stage	Processes	Identified signaling pathway components	Reference
**Early CNS development**	
	Check point for the progression of differentiation	*Ngn2*, *Pax6*	Fishwick et al., 2010
	NSCs size and switching cap-dependent translation on and off	S6K1/2, 4E-BPs	Hartman et al., 2013
	Neural progenitor development	GSK3, Sox2	Ka et al., 2014
	Soma size increase and axon extension	T3, BDNF, TrkB	Westerholz et al., 2013
	Impaired growth and interneuron migration and decreased survival	*Tsc1*	Fu et al., 2012
	Proliferation, survival, and neuronal differentiation	IGF1, TGFβ	Wahane et al. (2014)
	Neurite outgrowth	5HT7R, Erk, Cdk5, Cdc42 MeCbl Insulin, RAPTOR, RICTOR	Speranza et al., 2015 Okada et al., 2011 Urbanska et al., 2012
	Impaired neurite outgrowth and cell viability	Sirt1	Guo et al., 2011
	Oligodendrocyte development	Cannabinoid receptors Myelinating proteins: MBP, Tmem10, MOG, PLP	Gómez et al., 2011 Zou et al., 2014
**Adult CNS**	
• Hippocampus	Neurogenesis	Ezh2	Zhang et al., 2014
	Dendritic spine formation	IR, Rac *Disc1*	Lee et al., 2011 Kim et al., 2009
	Late-stage plasticity	HuD, CaMKIIα	Sosanya et al., 2015
	Enhanced spatial cognitive function	TrkA, ERK	Zhou et al., 2014
	Learning and memory performance	S6K1, 4E-BP1	Qi et al., 2010; Su et al., 2015
	Memory consolidation	Estrogen and progesterone, TrkB, ERK calpain BDNF, GluR1	Fortress et al., 2013; Briz and Baudry, 2014; Orr et al., 2012; Slipczuk et al., 2009
	Long-term memory, protein synthesis	Circadian oscillations eIF4E, 4E-BP1, S6, ERK1/2 CB1R	Saraf et al., 2014 Puighermanal et al., 2009
	Learning and memory deficits	DISC1	Zhou et al., 2013
• Striatum	Motor learning consolidation	S6K1, 4E-BP	Bergeron et al., 2014
• Amygdala	Memory consolidation of fear memory	KLK8, FKBP51	Jobim et al., 2012a,b; Pedroso et al., 2013 Hadamitzky et al., 2014 Mac Callum et al., 2014
• Medial prefrontal cortex	Trace fear memory Antidepressant effects	S6K1	Sui et al., 2008 Dwyer et al., 2015
• Auditory cortex	Memory consolidation for the discrimination of auditory stimuli	Dopaminergic activity	Schicknick et al., 2008
**CNS aging**	
	Increase lifespan	Decreased p-mTOR/mTOR	Triplett et al., 2015
	Decreased rate of degradation	Rag-Ragulator complex	Yu et al., 2014
	Decreased cognitive function	BDNF, Akt	Yang et al., 2014
	Learning and memory dysfunction	p62, S6K1	Dong et al., 2015a,b
	Alterations in histone methylation levels	H3K27me3, H3R2me2, H3K79me3 H3K4me2	Gong et al., 2015
	Enhanced autophagy, synaptic plasticity	CREB, decreased mTOR	Liu et al., 2015
	Better memory performance	*LARS2*, *FASTKD40*	Ramanan et al., 2015
	Adult neurogenesis	S6, EGF	Romine et al., 2015 Paliouras et al., 2012
	Circadian control of aging	BMAL1	Khapre et al., 2014

## Concluding Remarks

In early life mTOR participates in neurogenesis, in neurite outgrowth and elongation and finally in gliogenesis. All of these processes seem to be regulated by mTOR via translation, and it likely functions as a cell-cycle checkpoint. There is need of more *in vivo* studies that show the timing, region and cell specificity of mTOR activation/inhibition. It is also important to identified the signaling pathways and effectors that specifically participate in each these process.

In the adult brain, mTOR participates in key processes such as synaptic plasticity, adult neurogenesis, and learning and memory. Its role depends on many factors, and in some cases, it seems to have opposite actions. We also need to understand which mechanisms spatiotemporally modulate or balance mTOR expression and how their disruption is associated with neurodegenerative diseases. Addressing these gaps would help to generate therapeutic strategies.

Finally, during aging, mTOR seems to increase neurogenesis, decrease autophagy, and regulate epigenetic changes.

This kinase is an important energy sensor that is present throughout our lifespan. Several stimuli and transduction pathways tightly modulate its expression. Its role must be precisely described in order to develop therapeutic strategies and prevent CNS diseases.

## Author Contributions

All authors listed, have made substantial, direct and intellectual contribution to the work, and approved it for publication.

## Conflict of Interest Statement

The authors declare that the research was conducted in the absence of any commercial or financial relationships that could be construed as a potential conflict of interest.

## Abbreviations

4E-BP1: eukaryotic initiation factor 4E-binding protein
DEPTOR: DEP domain-containing mTOR-interacting protein
eEF2: eukaryotic elongation factor 2
eEF2K: eukaryotic elongation factor 2 kinase
eIF4E: eukaryotic translation initiation factor 4E
FKBP12: FK506-binding protein
mLST8: mammalian lethal with SEC13 protein 8
mPFC: medial prefrontal cortex
mSIN1: stress-activated MAP kinase-interacting protein 1
mTOR: mammalian target of rapamycin
mTORC1: mammalian target of rapamycin complex 1
mTORC2: mammalian target of rapamycin complex 2
NSCs: neural stem cells
PRAS40: 40 kDa Pro-rich AKT1 substrate 1
PROTOR: Pro-rich protein 5
RAPTOR: regulatory associated protein of mTOR
Rheb: Ras homolog enriched in brain
RICTOR: rapamycin-insensitive companion of mammalian target of rapamycin
S6K1: S6 kinase
SVZ: subventricular zone
TSC1: tuberous sclerosis complex proteins, hamartin
TSC2: tuberous sclerosis complex proteins, tuberin
